# Biomechanical model registration for monitoring and simulating large orthodontic tooth movements in the maxilla and mandible

**DOI:** 10.1007/s00056-022-00412-8

**Published:** 2022-07-08

**Authors:** Falko Schmidt, Fatih Kilic, Catrin Verena Gerhart, Bernd Georg Lapatki

**Affiliations:** Department of Orthodontics, Centre of Dentistry, Ulm University, Albert-Einstein-Allee 11, 89081 Ulm, Germany

**Keywords:** Diagnostic imaging, Model superimposition, Treatment simulation, Premolar extraction, Digital setup, Diagnostische Bildgebung, Modellüberlagerung, Behandlungssimulation, Prämolarenextraktion, Digitales Setup

## Abstract

**Purpose:**

Superimposition of digital dental-arch models allows quantification of orthodontic tooth movements (OTM). Currently, this procedure requires stable reference surfaces usually only present in the maxilla. This study aimed to investigate the accuracy of a novel superimposition approach based on biomechanical principles of OTM and the equilibrium of forces and moments (EFM)—applicable in both jaws—for monitoring and simulating large OTM.

**Methods:**

The study included 7 patients who had undergone extraction of the first (PM1-Ex) or second (PM2-Ex) premolar in each quadrant. Digital models taken at start and end of the T‑Loop treatment phase were superimposed by applying 3 EFM variants differing in the number of teeth used for registration. Maxillary OTM results for EFM were validated against those for a conventional surface registration method (SRM). In an additional case study, OTM were simulated for PM1-Ex, PM2-Ex and non-extraction treatment strategies.

**Results:**

The EFM variant that included all teeth of the dental arch achieved the highest accuracy, with median translational and rotational OTM deviations from SRM of only 0.37 mm and 0.56°, respectively. On average, retracted canines and first premolars were distalized by 3.0 mm, accompanied by 6.2° distal crown tipping and 12.2° distorotation. The share of space closure by molar mesialization was 19.4% for PM1-Ex quadrants and 34.5% for PM2-Ex quadrants.

**Conclusion:**

EFM allows accurate OTM quantification relative to the maxillary and mandibular bases even in challenging situations involving large OTM. Superimposition of malocclusion and setup models enables realistic simulation of final tooth positions. This may greatly enhance the value of digital setups for decision-making in orthodontic treatment planning.

## Abstract (de)

**Hintergrund:**

Die Überlagerung von digitalen Gebissmodellen ermöglicht die Quantifizierung von kieferorthopädischen Zahnbewegungen (OTM). Bisher erfordert dieses Verfahren stabile Referenzflächen, die zumeist nur im Oberkiefer vorhanden sind. Ziel dieser Studie war die Untersuchung der Genauigkeit einer neuartigen Überlagerungsmethode, die auf den biomechanischen Prinzipien von OTM und dem Gleichgewicht von Kräften und Momenten (EFM) basiert und in beiden Kiefern anwendbar ist, bei der Überwachung und Simulation großer OTM.

**Methoden:**

Die Studie umfasste 7 Patienten, bei denen der erste (PM1-Ex) oder zweite (PM2-Ex) Prämolar in jedem Quadranten extrahiert wurde. Digitale Modelle, erstellt zu Beginn und am Ende der T‑Loop-Behandlungsphase, wurden mit 3 verschiedenen EFM-Varianten überlagert, die sich in der Anzahl der für die Registrierung verwendeten Zähne unterscheiden. Die OTM-Ergebnisse im Oberkiefer wurden gegen eine konventionelle Oberflächenregistrierungsmethode (SRM) validiert. Zudem wurden in einer Fallstudie die OTM für die Behandlungsstrategien PM1-Ex, PM2-Ex und Nichtextraktion simuliert.

**Ergebnisse:**

Die EFM-Variante, die alle Zähne des Zahnbogens einbezog, erreichte die höchste Genauigkeit mit translatorischen und rotatorischen OTM-Abweichungen von der SRM von im Median nur 0,37 mm bzw. 0,56°. Die retrahierten Eckzähne bzw. ersten Prämolaren wurden im Mittel 3,0 mm distalisiert, begleitet von einer distalen Kronenkippung von 6,2° und einer Distorotation von 12,2°. Der Anteil des Lückenschlusses durch die Mesialisierung der Molaren betrug 19,4 % für PM1-Ex-Quadranten und 34,5 % für PM2-Ex-Quadranten.

**Schlussfolgerung:**

EFM ermöglicht eine genaue Quantifizierung der OTM relativ zur Ober- bzw. Unterkieferbasis selbst in schwierigen Situationen mit großen OTM. Die Überlagerung von Zahnfehlstellung und Setup-Modell ermöglicht eine realistische Simulation der finalen Zahnpositionen. Dadurch kann die Aussagekraft digitaler Setups für die Wahl der Behandlungsstrategie in der kieferorthopädischen Behandlungsplanung erheblich gesteigert werden.

## Introduction

Setups of the dental arches play a key role in orthodontic diagnostics and treatment planning [5]. After decades of use of plaster casts, application of digital dental-arch models for orthodontic diagnosis and virtual treatment planning has recently increased [8, 34]. Although commercial dental software packages provide digital setup tools, the underlying methods are unable to predict the final clinical dental arch position relative to the initial malocclusion and jaw base. This constitutes a major limitation because the actual position of the dental arch affects the occlusal relationships and the final incisor position influences the facial soft-tissue profile. This is particularly relevant if large orthodontic tooth movements (OTM) are required, as in premolar extraction.

In 1998, Alcañiz et al. [2] presented a concept for computer-aided orthodontic treatment simulation. Their analogical model for OTM has not, however, been validated. More sophisticated OTM simulations based on finite element analysis are usually applied for research purposes [19, 23]; however, time-consuming preprocessing and computation impede broad clinical application of such simulations in individual treatment cases. More efficient and realistic approaches for visualization and quantification of therapeutically required OTM may rely on superimposition of pretreatment and virtual setup models. Previous approaches [7, 28] registered models via surface alignment at the tooth crowns, which either limits applicability to very small OTM [7, 13] or disregards concomitant movement of reference teeth due to anchorage loss [20, 33] and physiological drift of teeth [27]. The frequently proposed surface alignment of jaw models in relation to attached soft tissue such as the hard palate and palatal rugae [9, 10, 18, 22, 36, 38] is only applicable to the maxilla. In the mandible, only the (rarely available) mandibular tori provide a reliable reference [3]. The use of mini-implants as markers [9, 15] is invasive and, therefore, not applicable in clinical routine. A general limitation of structures further away from the teeth [14, 17, 25] is related to morphological changes over time [29, 31], which not only occur during tooth eruption and the main growth period, but also in adults [11, 35]. Generally, treatment simulation should primarily focus on treatment-induced changes. Other factors such as growth should be considered separately.

We recently introduced a novel biomechanical approach for dental-arch model superimposition [31], called “equilibrium of forces and moments” (EFM), for OTM monitoring during the levelling and alignment phase of fixed appliance therapy. Results were validated against several established surface registration methods (SRM) using palatal soft-tissue regions. The present study aims to investigate the applicability of EFM for monitoring and simulating premolar extraction treatments that implement large OTM. First, OTM during the space-closure phase of clinical cases was determined by EFM. Accuracy was validated against results from conventional SRM in the maxilla. Second, a case study demonstrates how EFM may be used for simulation and decision-making in treatment planning.

## Materials and methods

### Patient records

**Fig. 1:**
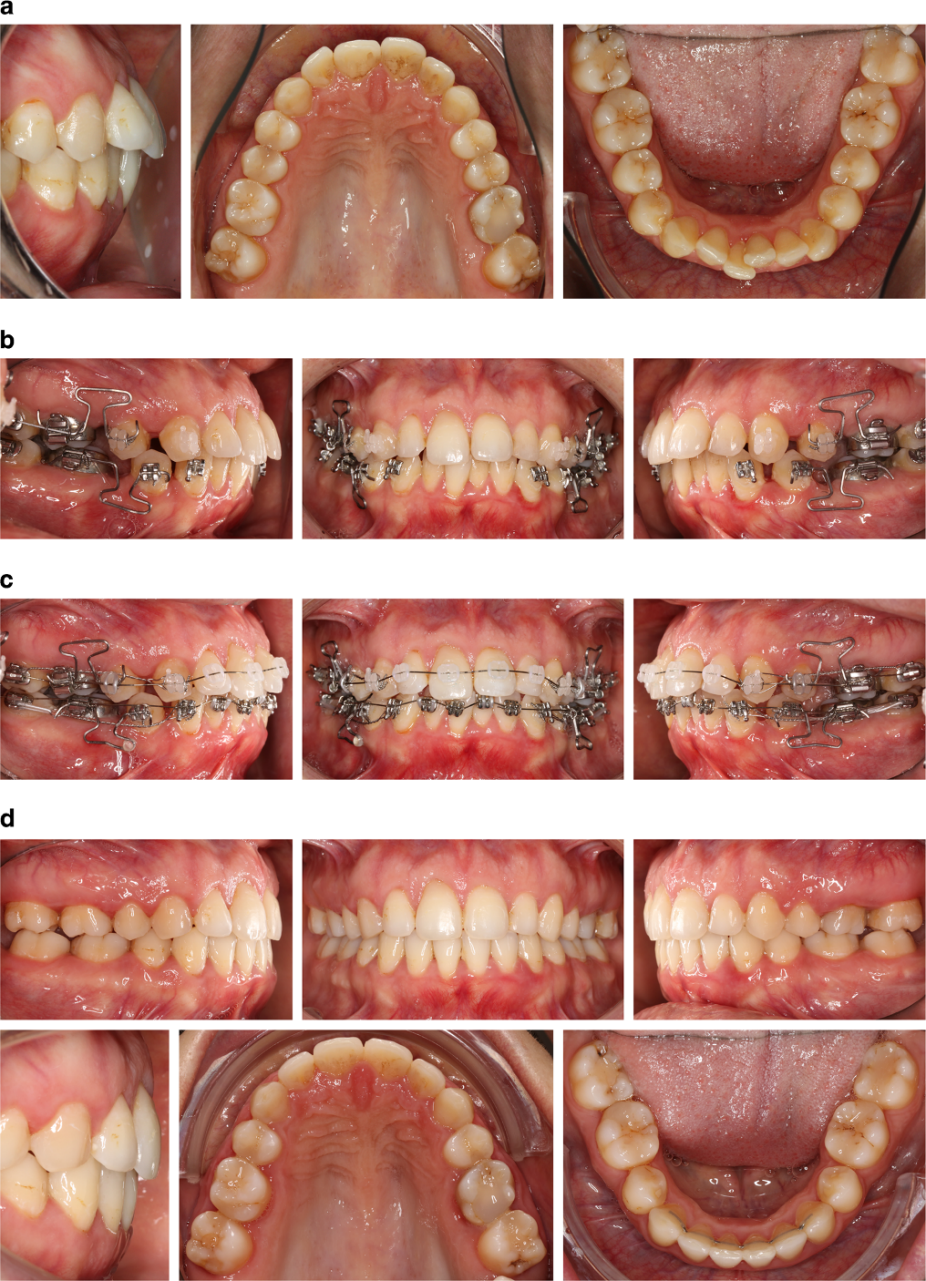
Intraoral photographs of a 33-year-old woman who was presented in the case study for treatment simulation. **a** Pretreatment situation. **b** Partial space closure achieved after 4 months using segmental T‑Loop wires in the posterior segments. **c** Begin of levelling and alignment phase 6 months after start of treatment. **d** Treatment outcome after 13 months Intraoralfotos einer 33-jährigen Patientin, die in der Fallstudie zur Behandlungssimulation vorgestellt wurde. **a** Situation vor der Behandlung. **b** Teilweiser Lückenschluss nach 4 Monaten mit Teilbogen mit T‑Loop im Seitenzahnbereich. **c** Beginn der Nivellierungs- und Ausrichtungsphase 6 Monate nach Beginn der Behandlung. **d** Behandlungsergebnis nach 13 Monaten

This study used actual treatment results from the first 7 patients (6 female, 1 male; aged 16–43 years at treatment start) of a larger prospective trial approved by the Ethics Committee of Ulm University (no. 303/20); all patients provided written informed consent. Included patients had severe anterior crowding requiring extraction of one premolar per quadrant and all teeth attached to the appliance had to be fully erupted (see Fig. 1 for intraoral photographs before, during and after orthodontic treatment for an exemplary patient). Extraction spaces were closed using one 0.016 × 0.016-inch T‑Loop wire per quadrant which excluded the anterior teeth (Fig. 1b). The average duration of the T‑Loop treatment phase was 4.7 months (range 2.4–7.6 months).

### Data acquisition and processing

**Fig. 2:**
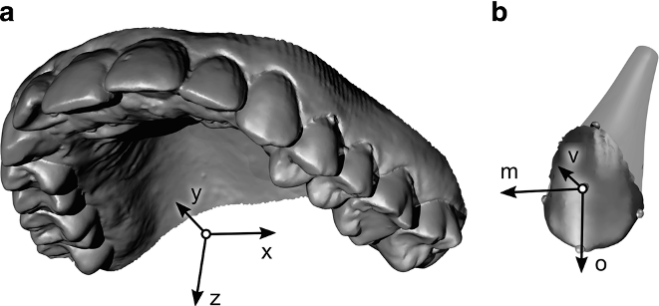
Coordinate systems used to describe orthodontic tooth movement (OTM). **a** Jaw coordinate system. The x,y-plane coincides with the occlusal plane, and the z‑axis points towards the opposite jaw. The x‑axis was defined by the distobuccal cusps of the first molars pointing to the patient’s left side in the maxilla and to the right side in the mandible. The y‑axis points in the anterior direction toward the point of contact of the central incisors. **b** Local tooth coordinate system shown on the crown of a left upper canine with a schematic tooth root for illustration. The origin is located at the center of the visible vestibular crown surface, i.e., the load application point corresponding to the center of an attached bracket. *Spheres* indicate landmarks used to define the orientation of the axis system. The occlusal direction (o) was set up to respect typical angulation and inclination [4] to the clinical axis of the crown, which was defined by center points at the incisal edge and vestibular gum line. The mesial direction (m) was determined using the distal and mesial landmarks at the crown, and the vestibular (v) direction points toward the lip or cheeks. This definition implies use of right-handed (first and third quadrant) as well as left-handed (second and fourth quadrant) coordinate systems Koordinatensysteme, die zur Beschreibung der kieferorthopädischen Zahnbewegung (OTM) verwendet werden. **a** Kieferkoordinatensystem. Die xy-Ebene liegt in der Okklusionsebene, die z‑Achse zeigt in Richtung des Gegenkiefers. Die x‑Achse wurde durch die distobukkalen Höcker der ersten Molaren definiert. Sie zeigt im Oberkiefer zur linken Seite des Patienten und im Unterkiefer zur rechten Seite. Die y‑Achse zeigt nach anterior zum Kontaktpunkt der mittleren Schneidezähne. **b** Lokales Zahnkoordinatensystem, dargestellt an der Krone eines linken oberen Eckzahns mit einer schematischen Zahnwurzel. Der Ursprung liegt in der Mitte der sichtbaren vestibulären Kronenfläche, d. h. im theoretische Lastangriffspunkt in der Mitte eines angebrachten Brackets. Die *Kugeln* zeigen Orientierungspunkte zur Ausrichtung des Achsensystems. Die Lage der okklusalen Richtung (o) zur Zahnkronenachse (definiert durch die Mittelpunkte der Inzisalkante und dem vestibulären Zahnfleischrand) berücksichtigt eine typische Angulationen und Neigungen [4] des Zahns. Die mesiale Richtung (m) wurde durch die distalen und mesialen Orientierungspunkte an der Krone bestimmt, die vestibuläre Richtung (v) zeigt in Richtung Lippe bzw. Wange. Diese Definition impliziert die Verwendung sowohl rechtshändiger (erster und dritter Quadrant) als auch linkshändiger (zweiter und vierter Quadrant) Koordinatensysteme

Three-dimensional (3D) digital full-arch models were obtained at T0 (before bracketing) and T1 (before insertion of the first full archwire) using an intraoral scanner (TRIOS 3, 3Shape, Copenhagen, Denmark). Individual tooth crowns were separated from T0 models, and the centers of the visible labial crown surfaces were localized using dental imaging software (OnyxCeph, Image Instruments, Chemnitz, Germany). Second molars were only considered if they were completely erupted at T0. Data were further processed using OptoCat software (AICON 3D Systems, Braunschweig, Germany). After definition of a jaw coordinate system (Fig. 2a), the separated tooth crowns were superimposed with their analogue counterparts on both the T0 and T1 models using a closest point algorithm.

### EFM for determining OTM in maxilla and mandible

In accordance with Newton’s third axiom, the underlying biomechanical OTM model is based on the equilibrium of forces and moments acting on the individual teeth due to the activation of the orthodontic appliance. This assumption is widely accepted and validated in orthodontics [24]. Principally, the method determines the spatial interrelation between two models in which the differences of corresponding 3D positions of the individual teeth (i.e., OTM during treatment) are best explained by the balance equations of the forces and moments that induced these OTMs. In order to estimate the loads acting on each individual tooth from their positional changes, the movement was considered to be continuous and uniform from their initial to final positions. A constant remodeling rate for resorption and apposition was implemented driven by a stress stimulus in the PDL derived using Hooke’s law and a simplified representation of the tooth root morphologies that build upon morphological data from the literature. It is important to note that all loads contributing to the OTMs are relevant to the EFM approach, even if they are not directly transmitted by the orthodontic appliance. The numerical equations implemented were described in detail previously [31]. As an evolution of our previous model, individual tooth coordinate systems were defined independently of adjacent teeth (Fig. 2b).

Since the equilibrium of forces and moments requires the consideration of all loads acting on a system, the system boundaries were questioned and 3 different EFM variants were compared:

- EFM1: only teeth connected to the appliance were considered, based on the assumption that solely the periodontal ligaments of these teeth support orthodontic loads.
- EFM2: as an extension of EFM1, the two teeth adjacent to the appliance were also considered because loads can partly be transferred to neighboring teeth via proximal contacts and transseptal fibers.
- EFM3: all teeth of the dental arch were considered. Because the anterior teeth were not included in the appliance, their position during treatment may be assumed to be stable; hence, their involvement may stabilize the optimization algorithm.

### Validation of EFM for maxillary OTM

For validation, the numerical EFM superimposition algorithm was replaced by a surface registration method (SRM) proposed by Choi et al. [10]. The reference region comprised the entire hard palate with lateral boundaries approximately 5 mm from the gingival margins, excluding the incisive papilla. As this method relies on the palatal vault, its application was limited to the upper jaw. Each patient was evaluated only once because interoperator variability of both methods was already shown to be small [31].

### Comparison of OTMs of different treatment options in a case study

EFM allows superimposition of digital dental models based on the changes in position of the teeth independently of other reference structures. Thus, it can be used with real scans to determine OTMs achieved, but also with digital setup models to determine the OTMs required for a desired treatment outcome. In this case study, EFM was used to determine OTMs for different treatment options for one finished treatment case with a 2.0-mm space discrepancy and moderate incisor proclination (+8°) in the maxillary arch, and a 4.8-mm space discrepancy combined with pronounced incisor proclination (+15°) in the mandibular arch (Fig. 1).

Three alternative treatment options were considered: (a) extraction of the four first premolars (PM1-Ex), (b) extraction of the four second premolars (PM2-Ex), and (c) treatment without extraction (Non-Ex). For each treatment option, an experienced orthodontist performed digital setups of the maxillary and mandibular dental arches with corrected individual tooth positions and closed extraction spaces using a standard dental image-processing software (OnyxCeph). These target setup models were then superimposed with the initial malocclusion models using EFM3 to calculate the final positions of the maxillary and mandibular dental arches and the corresponding OTM for individual teeth. For comparison, EFM3 was also used to quantify OTM for the actual treatment.

For the two extraction treatment options, we also investigated whether the extractions provide sufficient space for correcting the malpositions within the anterior segment. For this purpose, we simulated the OTMs (of the individual teeth) from alignment of the anterior teeth independently. To achieve this, we superimposed the T0 models with the digital setups of the intended posttreatment dental arches by applying EFM solely to the teeth anterior to the extraction spaces. The difference between the initial extraction-space width and distally directed OTM derived for the tooth located mesially (i.e., the canine or first premolar) indicate whether space remains after extractions and anterior corrections.

## Results

### Three-dimensional OTM in maxillary and mandibular arches

**Fig. 3:**
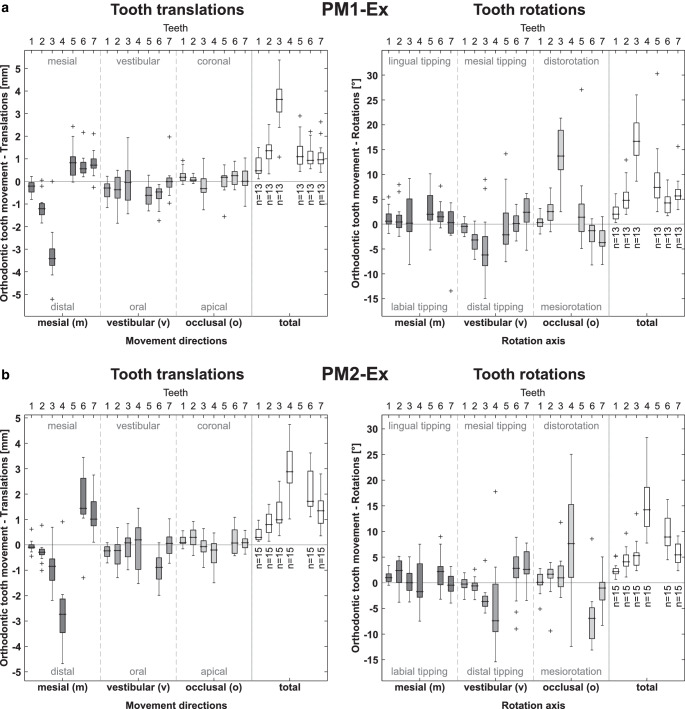
Orthodontic tooth movement derived for quadrants with **a** first (PM1-Ex) and **b** second (PM2-Ex) premolar extraction cases, determined by the third variant of the equilibrium of forces and moment method (EFM3). Translational (*left*) and rotational (*right*) movements refer to the center of the clinically visible vestibular crown center (Fig. 2b), which corresponds to the orthodontic load application point. Results are pooled for right and left as well as maxillary and mandibular quadrants. Tooth numbering: *1* central incisor, *2* lateral incisor, *3* canine, *4* first premolar, *5* second premolar, *6* first molar, and *7* second molar Kieferorthopädische Zahnbewegungen für Quadranten mit Extraktionsfällen der **a** ersten (PM1-Ex) und **b** zweiten (PM2-Ex) Prämolaren, ermittelt mit der dritten Variante der Kräfte- und Momente-Gleichgewicht-Methode (EFM3). Translations- (*links*) und Rotationsbewegungen (*rechts*) beziehen sich auf das Zentrum der klinisch sichtbaren vestibulären Kronenmitte (Fig. 2b), was dem kieferorthopädischen Lastangriffspunkt entspricht. Die Ergebnisse sind sowohl für rechte und linke Quadranten als auch für Ober- und Unterkiefer zusammengefasst. Nummerierung der Zähne: *1* mittlere Schneidezähne, *2* seitliche Schneidezähne, *3* Eckzähne, *4* erste Prämolaren, *5* zweite Prämolaren, *6* erste Molaren und *7* zweite Molaren

OTMs from actual treatment outcomes determined by EFM3 are depicted in Fig. 3. Maxillary and mandibular teeth are pooled. Regarding translational OTMs, distalization of the canine (median value:−3.42 mm) significantly exceeded mesialization of the second premolar (+0.82 mm) and first molar (+0.65 mm) in the first-premolar extraction case (Fig. 3a). This corresponds to a space-closure ratio of 80.6% and 19.4% from the mesial and distal sides, respectively. Canine distalization was accompanied by 6.2° distal tipping and 13.7° distorotation (Fig. 3a). Rotational components for the second premolar and first molar were small. The lateral incisor (not integrated in the T‑Loop mechanics) was passively distalized by 1.21 mm. In quadrants with second-premolar extraction (Fig. 3b), median values for distalization of the first premolar and mesialization of the first molar were−2.73 mm (65.5%) and 1.44 mm (34.5%), respectively. Again, collateral distal tipping (−7.41°) and distorotation (7.63°) were observed for the retracted tooth. Median passive distal movement of the canine was−0.85 mm.

### Validation of EFM results for maxillary OTM

**Fig. 4:**
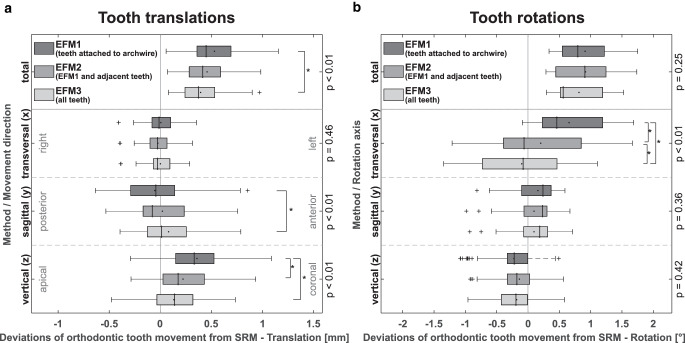
Deviation of orthodontic tooth movement (OTM) from the reference, i.e., OTM obtained by the surface registration method (SRM) proposed by Choi et al. [10], as determined by 3 investigated equilibrium of forces and moments method (EFM) variants. Results include total deviations and directional components for tooth translations (**a**) and rotations (**b**) in reference to the centers of the vestibular crown surfaces of individual teeth in the jaw coordinate system (Fig. 2a). In each *box*, the results for all upper teeth of 7 patients are combined (*n* = 84). The *p*-values were obtained from Friedman’s test that accounts for any statistically significant difference among all 3 variants. *Brackets* indicate statistically significant differences (* *p* < 0.05) for pairwise comparison derived from post hoc analysis [12] Abweichung der kieferorthopädischen Zahnbewegung (OTM) für 3 untersuchte Varianten der Kräfte- und Momente-Gleichgewicht-Methode (EFM) von der Referenz, für welche die Oberflächenregistrierungsmethode (SRM) nach Choi et al. [10] verwendet wurde. Die Ergebnisse umfassen Gesamtabweichungen und Richtungskomponenten für Zahntranslationen (**a**) und -rotationen (**b**) in Bezug auf die Zentren der vestibulären Kronenoberflächen der einzelnen Zähne im Kieferkoordinatensystem (Fig. 2a). In jeder *Box* sind die Ergebnisse für alle oberen Zähne von 7 Patienten zusammengefasst (*n* = 84). Die *p*-Werte wurden mit dem Friedman-Test ermittelt, der jeden statistisch signifikanten Unterschied zwischen den 3 Varianten berücksichtigt. *Klammern* kennzeichnen statistisch signifikante Unterschiede (* *p* < 0,05) für den paarweisen Vergleich der Post-hoc-Analyse [12]

Fig. 4 shows translational and rotational deviations between maxillary OTMs determined by SRM and corresponding results found by the EFM variants. The representation in jaw coordinates reflects 3D positional discrepancies in the registration of the complete dental-arch models. Regarding total translations, median deviations from SRM results were significantly smaller for EFM3 (0.37 mm) than for EFM1 (0.45 mm; Friedman’s test, *p* < 0.01 and Conover post hoc test), with a maximal deviation of 1.15 mm for EFM1. Results for spatial components show that deviations between EFM and SRM predominantly relate to vertical discrepancies and indicate relative supraposition of the teeth for EFM superimposition. Generally, OTMs determined by the 3 EFM variants show small rotational deviations from SRM results, with median values between 0.56° (EFM3) and 0.91° (EFM2) and a maximum of 1.75° (EFM1). Here, maximal deviations are observed for rotations about the transversal axis, with significant differences between the EFM variants (*p* < 0.01).

### Treatment simulation vs. actual treatment

The simulated positions of incisors and first molars after complete correction are given in Table 1 for the 3 treatment options (PM1-Ex, PM2-Ex, Non-Ex). In the maxilla, simulations predict larger lingual displacements of the central incisor crowns for PM1-Ex (right 4.75 mm, left 5.42 mm) than for PM2-Ex (right 3.35 mm, left 3.90 mm). The latter values showed good agreement with the corresponding actual treatment results (right 3.50 mm, left 4.56 mm). For mesialization of the upper first molars, deviations between PM2-Ex and actual treatment were also relatively small (right 0.57 mm, left 0.37 mm). In the mandible, labial displacements of the right (1.55 mm) and left (3.02 mm) central incisors were predicted for Non-Ex. For the simulated extraction treatments, in contrast, movement of these teeth was lingual, with larger values for PM1-Ex (right 3.27 mm, left 1.72 mm) than for PM2-Ex (right 2.38 mm, left 0.61 mm). The PM2-Ex values differed from the corresponding clinical results by 0.32 mm (right) and 0.80 mm (left). Prediction of lower first molar mesialization showed similar accuracy with deviations of 0.63 mm (right) and 0.27 mm (left).

**Table 1:** Orthodontic tooth movements of individual teeth for different treatment options Kieferorthopädische Zahnbewegungen einzelner Zähne für verschiedene Behandlungsoptionen

	Simulation/Treatment case	Teeth extracted	Teeth retracted in loop phase	Simulated correction of anterior segment	After completion of treatment		
				Distal OTM of retracted tooth (mm)	Distal OTM of retracted tooth (mm)	Mesial OTM of first molar (mm)	Lingual OTM of central incisor (mm)
				Q1/Q2	Q1/Q2	Q1/Q2	Q1/Q2
Maxilla	Non-Ex	–/–	–/–	–/–	–/–	−0.19/−0.06	−0.07/0.01
	PM1-Ex	14/24	13/23	2.44/2.77	4.65/4.88	2.18/1.42	4.75/5.42
	*PM2-Ex*^*a*^	*15/25*	*14/24*	*2.61/2.51*	*4.84/4.05*	*2.78/2.43*	*3.35/3.90*
	*Treatment outcome*^*a*^	*15/25*	*14/24*	*–/–*	*4.28/3.22*	*3.35/2.80*	*3.50/4.56*
				Q4/Q3	Q4/Q3	Q4/Q3	Q4/Q3
Mandible	Non-Ex	–/–	–/–	–/–	–/–	0.23/0.11	−1.55/−3.02
	PM1-Ex	34/44	33/43	1.23/2.39	3.33/4.35	2.47/2.55	3.27/1.72
	*PM2-Ex*^*a*^	*35/45*	*34/44*	*2.17/2.87*	*4.00/4.31*	*4.03/3.25*	*2.38/0.61*
	*Treatment outcome*^*a*^	*35/45*	*34/44*	*–/–*	*4.07/4.11*	*3.40/2.98*	*2.70/1.41*

Orthodontic tooth movements (OTM) of the tooth crowns derived for different treatment approaches, i.e., extraction of the first (PM1-Ex) or second (PM2-Ex) premolars as well as without tooth extraction (Non-Ex).

OTM values refer to the centers of the vestibular crown surfaces of individual teeth and are determined for each quadrant individually with respect to the situation before treatment start (T0). *Q1, Q2, Q3* and *Q4* refer to the upper right, upper left, lower left and lower right quadrant, respectively

^a^Simulation results are contrasted with actual treatment outcome (italic font) of a patient with crowding in the upper and lower dental arches. PM2-Ex (italic font) corresponds to the treatment strategy chosen

In the context of PM2-Ex treatment simulation, isolated simulation of the correction of the eight anterior teeth resulted in distal movement of the four first premolars in the range of 2.17–2.87 mm. In comparison, after simulation of complete space closure, first premolars were distalized by 4.00–4.84 mm in total (Table 1). This indicates that PM2-Ex provided sufficient space in all four quadrants. Table 1 also provides corresponding results for PM1-Ex treatment simulations.

## Discussion

The investigated method for dental-arch model superimposition called EFM is based on biomechanical principles and allows monitoring and simulation of effective 3D OTMs of individual teeth within subgroups of teeth as well as the complete dental arch. An important precondition for EFM is that the orthodontic appliance does only have an effect on teeth within the same dental arch, without any support on the opposite arch or external structures such as orthodontic mini-implants. Unlike EFM, conventional superimposition approaches depend on stable gingival or dental surfaces or both. Stable gingival surfaces with sufficient distance from the tooth crowns are predominantly found in the hard palate. In the mandible, such structures are only rarely available. Hence, the application of gingivae-dependent methods is usually restricted to the maxillary arch. Conventional superimposition methods that purely rely on dental surfaces may theoretically be applied to both arches. Orthodontic therapy, however, usually results in movement of all teeth of the respective dental arch because, even for large anchorage units, reactive forces and moments induce at least minor reactive OTM [26, 37]. Moreover, even teeth which are not included in the appliance show passive OTM, e.g., due to load transmission via soft-tissue structures and interproximal contacts (also apparent in our results; Fig. 3). Hence, the registration of dental-arch models from different treatment stages purely based on superimposition of dental surfaces will arguably always be compromised.

For validation, OTMs derived from 3 EFM variants were compared with those obtained from conventional SRM using palatal surface registration. Previous studies [6, 9] found significant changes in the position of the first two palatine rugae during treatment of extraction cases and, consequently, proposed omission of this region for SRM. Nevertheless, we used the entire palatal vault surface for superimposition, to increase registration reliability [31]. This was reasonable because the monitored treatment period ended before the retraction of anterior teeth might have induced changes in the rugal region.

Generally, OTM values derived from the investigated EFM strategies deviated only slightly from the SRM results. This particularly concerns the 3 rotational components (median deviations < 0.5°) as well as the transversal and sagittal translations (median deviations < 0.08 mm). Considering the large translational movements observed for the retracted teeth, with median values > 3 mm, these small deviations may be considered clinically negligible. One might speculate that the somewhat larger, yet clinically insignificant deviations between EFM and SRM observed for vertical translations (median for EFM3: 0.14 mm) are related to the considerable tipping movements of adjacent teeth into the extraction space, leading to occlusal precontacts which might have induced intrusive forces. The EFM method does not take the latter into account. Another explanation might be the imbalanced sex distribution of the evaluated patients. Morphological data from literature used in EFM may overestimate the root size of women [21], which, in turn, may overestimate the resistance of tipping teeth against vertical movements. We therefore suppose that among the simplifications introduced in the EFM model [31], the implementation of average morphological data is a major source of remaining inaccuracies of EFM model superimposition. This limitation could be addressed by individualization of tooth geometries, e.g., on the basis of panoramic radiographs [30].

The greater accuracy in EFM2 and even greater accuracy in EFM3 indicates that EFM superimposition gains stability from including teeth that are not involved in the appliance. However, in clinical treatment, the largest OTM usually occur in the sagittal and transversal directions, where EFM provided very accurate results (Fig. 3). Since there is no apparent reason why the application of EFM should be more inaccurate in the mandible than in the maxilla, one might extrapolate the remarkably high accuracy demonstrated for maxillary OTM to mandibular OTM.

The separate evaluation of OTM for quadrants with PM1-Ex and PM2-Ex exemplifies the great clinical potential of dental-arch model superimposition based on EFM. It allows quantification of mesial and distal shares of OTM required for space closure. Assuming that an extracted premolar provides approximately 6.7 mm [32] space in the dental arch, space-closure ratios of 80.6% (PM1-Ex) and 65.5% (PM2-Ex) mean that extraction of the first or second premolar would provide approximately 5.4 mm or 4.4 mm space per quadrant, respectively, for alignment and uprighting of the frontal segment. These values are relevant for helping clinicians to decide between both extraction options. Another clinically interesting finding was a significant tipping and rotation of the first molars, canines and first premolars into the extraction space. Counter-tip (15°) and counter-rotation (30°) preactivations of the T‑Loop wires were apparently insufficient to compensate for these concomitant collateral movements. Furthermore, the quantitative information provided by this study regarding passive OTM of anterior teeth excluded from the appliance (a well-known phenomenon during extraction-space closure or other large OTMs) is of clinical interest.

As demonstrated by our case study, EFM may be applied both retrospectively to quantify OTM after orthodontic treatment, or prospectively to simulate treatment. Regarding the latter, application of EFM may considerably improve the accuracy and informative value of diagnostic setups because feasibility and treatment effort can be assessed more realistically. In a conventional setup procedure, positioning of individual teeth and tooth segments with respect to the dentoalveolar base is somewhat arbitrary. Especially the chosen anteroposterior position of teeth may vary considerably between clinicians due to the lack of suitable references. In contrast, superimposition of the malocclusion and setup models using EFM interrelates the dental arches according to objective and widely accepted biomechanical criteria. The added value of such EFM-aided treatment planning is exemplified by the presented case study results.

First, superimposition of the pretreatment models with setup models reflecting the treatment goal enabled prediction of total therapeutic OTM. It is noteworthy that such target setups may easily be revised to run through alternative treatment scenarios. An obvious benefit of such simulations is an estimation of the incisors’ final positions relative to the jaws and facial soft-tissues. Such information is of utmost importance in treatment planning because these positions substantially affect esthetic and functional outcomes as well as treatment stability [1, 16]. The variation of the sagittal changes of the central incisors’ positions for the 3 simulated treatment options in the maxilla (range: 1.0 to 5.1 mm) and mandible (range:−1.9 to 2.5 mm) indicate the considerable potential influence of sagittal changes on the lip profile because the lip contour follows 70–80% of these changes [16]. With respect to the sagittal central incisor position for PM2-Ex, the actual treatment outcome differed from the simulation by only 0.48 mm on average, which can be considered reasonably accurate. Another clinically highly relevant benefit of EFM-aided treatment simulation is the prediction of differential sagittal OTM of posterior teeth in the maxillary and mandibular arches. Such data may reveal the influence of different therapeutic strategies on the occlusal relationship. Unsatisfactory results after EFM superimposition may indicate the need for additional skeletal or intermaxillary anchorage to achieve neutral occlusion in the buccal segments. Such considerations are part of most orthodontic treatments.

The included simulation study further demonstrates that particularly in premolar extraction cases, further applications of EFM-aided treatment simulation are conceivable. This not only concerns the comparison of the provided and required space after premolar extraction (as exemplified here), but also the optimal timing of inclusion of the front teeth in the appliance or deactivation of the T‑Loop once the retracted tooth has reached its final position. Detailed explanation of these options, however, are beyond the scope of this paper.

Besides the predictive accuracy of the superimposition method, treatment simulations based on orthodontic setups are fundamentally limited by the degree of agreement between simulated and therapeutically achieved tooth positions, which mainly depends on realistic planning and the professional skills of the orthodontist. This particularly applies for sagittal root positions and inclinations of the incisors, which substantially affect the final anteroposterior position of the dental arch.

## Conclusion

EFM-based dental-arch model superimposition relies on biomechanical principles of OTM. Its application presumes that the orthodontic appliance is having an effect purely on the teeth of the corresponding dental arch without any additional external mechanical support. Beyond retrospective monitoring of therapeutic OTM, the method can also be applied for simulation of OTM to facilitate decision-making in clinical treatment planning. Because it can be used for both the maxillary and mandibular arches, and achieves high accuracy even for challenging situations such as premolar extractions, the EFM approach may be regarded as a major step forward in orthodontic treatment monitoring and simulation.

## References

[1] Ackerman JL, Proffit WR, Sarver DM (1999). The emerging soft tissue paradigm in orthodontic diagnosis and treatment planning. Clin Orthod Res.

[2] Alcañiz M, Montserrat C, Grau V (1998). An advanced system for the simulation and planning of orthodontic treatment. Med Image Anal.

[3] An K, Jang I, Choi D-S (2015). Identification of a stable reference area for superimposing mandibular digital models. J Orofac Orthop.

[4] Andrews LF (1989). Straight Wire. The concept and appliance.

[5] de Araújo TM, Fonseca LM, Caldas LD (2012). Preparation and evaluation of orthodontic setup. Dental Press J Orthod.

[6] Bailey LT, Esmailnejad A, Almeida MA (1996). Stability of the palatal rugae as landmarks for analysis of dental casts in extraction and nonextraction cases. Angle Orthod.

[7] Camardella LT, Rothier EKC, Vilella OV (2016). Virtuelles Setup: Anwendung in der kieferorthopädischen Praxis (Virtual setup: application in orthodontic practice). J Orofac Orthop.

[8] Carvalho PEG, Ortega AO, Maeda FA (2019). Digital Scanning in Modern Orthodontics. Curr Oral Health Rep.

[9] Chen G, Chen S, Zhang XY (2011). Stable region for maxillary dental cast superimposition in adults, studied with the aid of stable miniscrews. Orthod Craniofac Res.

[10] Choi D-S, Jeong Y-M, Jang I (2010). Accuracy and reliability of palatal superimposition of three-dimensional digital models. Angle Orthod.

[11] Christou P, Kiliaridis S (2008). Vertical growth-related changes in the positions of palatal rugae and maxillary incisors. Am J Orthod Dentofacial Orthop.

[12] Conover WJ (1999). Practical nonparametric statistics.

[13] Grauer D, Proffit WR (2011). Accuracy in tooth positioning with a fully customized lingual orthodontic appliance. Am J Orthod Dentofacial Orthop.

[14] Han G, Li J, Wang S (2019). In-vitro assessment of the accuracy and reliability of mandibular dental model superimposition based on voxel-based cone-beam computed tomography registration. Korean J Orthod.

[15] Hayashi K, Uechi J, Murata M (2004). Comparison of maxillary canine retraction with sliding mechanics and a retraction spring: a three-dimensional analysis based on a midpalatal orthodontic implant. Eur J Orthod.

[16] Hodgkinson D, Firth FA, Farella M (2019). Effect of incisor retraction on facial aesthetics. J Orthod.

[17] Hwang M, Ahn H-W, Kwon S-Y (2018). Control of anterior segment using an antero-posterior lingual sliding retraction system: a preliminary cone-beam CT study. Prog Orthod.

[18] Keilig L, Piesche K, Jäger A (2003). Applications of surface-surface matching algorithms for determination of orthodontic tooth movements. Comput Methods Biomech Biomed Engin.

[19] Kojima Y, Fukui H (2012). Numerical simulations of canine retraction with T-loop springs based on the updated moment-to-force ratio. Eur J Orthod.

[20] Kravitz ND, Kusnoto B, BeGole E (2009). How well does Invisalign work? A prospective clinical study evaluating the efficacy of tooth movement with Invisalign. Am J Orthod Dentofacial Orthop.

[21] Kulkarni V, Duruel O, Ataman-Duruel ET (2020). In-depth morphological evaluation of tooth anatomic lengths with root canal configurations using cone beam computed tomography in North American population. J Appl Oral Sci.

[22] Li S, Xia Z, Liu SS-Y (2015). Three-dimensional canine displacement patterns in response to translation and controlled tipping retraction strategies. Angle Orthod.

[23] Likitmongkolsakul U, Smithmaitrie P, Samruajbenjakun B (2018). Development and validation of 3D finite element models for prediction of orthodontic tooth movement. Int J Dent.

[24] Lindauer SJ (2001). The basics of orthodontic mechanics. Semin Orthod.

[25] Park T-J, Lee S-H, Lee K-S (2012). A method for mandibular dental arch superimposition using 3D cone beam CT and orthodontic 3D digital model. Korean J Orthod.

[26] Ren Y, Maltha JC, van ’t Hof MA (2004). Optimum force magnitude for orthodontic tooth movement: a mathematic model. Am J Orthod Dentofacial Orthop.

[27] Roberts WE (2000). Bone physiology of tooth movement, ankylosis, and osseointegration. Semin Orthod.

[28] Rodrigues MAF, Silva WB, Barbosa Neto ME (2007). An interactive simulation system for training and treatment planning in orthodontics. Comput Graph.

[29] de Oliveira Ruellas AC, Huanca Ghislanzoni LT, Gomes MR (2016). Comparison and reproducibility of 2 regions of reference for maxillary regional registration with cone-beam computed tomography. Am J Orthod Dentofacial Orthop.

[30] Schmidt F, Geiger ME, Jäger R (2016). Comparison of methods to determine the centre of resistance of teeth. Comput Methods Biomech Biomed Engin.

[31] Schmidt F, Kilic F, Piro NE (2018). Novel method for superposing 3D digital models for monitoring orthodontic tooth movement. Ann Biomed Eng.

[32] Schumacher G-H, Schmidt H (1983). Anatomie und Biochemie der Zähne.

[33] Simon M, Keilig L, Schwarze J (2014). Treatment outcome and efficacy of an aligner technique—regarding incisor torque, premolar derotation and molar distalization. BMC Oral Health.

[34] Tarraf NE, Ali DM (2018). Present and the future of digital orthodontics. Semin Orthod.

[35] Thilander B (2009). Dentoalveolar development in subjects with normal occlusion. A longitudinal study between the ages of 5 and 31 years. Eur J Orthod.

[36] Thiruvenkatachari B, Al-Abdallah M, Akram NC (2009). Measuring 3-dimensional tooth movement with a 3-dimensional surface laser scanner. Am J Orthod Dentofacial Orthop.

[37] van Leeuwen EJ, Kuijpers-Jagtman AM, den von Hoff JW (2010). Rate of orthodontic tooth movement after changing the force magnitude: an experimental study in beagle dogs. Orthod Craniofac Res.

[38] Vasilakos G, Schilling R, Halazonetis D (2017). Assessment of different techniques for 3D superimposition of serial digital maxillary dental casts on palatal structures. Sci Rep.

